# The comparison of species diversity and abundance of insect natural enemies in the domesticated species of cotton using the yellow pan trap method

**DOI:** 10.1038/s41598-023-48347-3

**Published:** 2024-02-02

**Authors:** Prabhulinga Tenguri, Sandhya Kranthi, Chinna Babu Naik, Amutha Mari, Rishi Kumar, Ruchika Suke, Vishlesh Shankar Nagrare, Nandini Gokte Narkhedkar, Vijay Namdeo Waghmare, Yenumula Gerard Prasad

**Affiliations:** 1 ICAR - Central Institute for Cotton Research, Postbag No 2, Shankar Nagar PO, Nagpur, Maharashtra 440010 India; 2 International Cotton Advisory Committee (ICAC), 1629, K Street, NW, Suite 702, Washington, DC 20006 USA; 3 ICAR - Central Institute for Cotton Research, Regional Station, Coimbatore, Tamil Nadu 641003 India; 4 ICAR - Central Institute for Cotton Research, Regional Station, Sirsa, Haryana 125055 India

**Keywords:** Ecology, Zoology

## Abstract

India is the world’s largest cotton producer and the only country that grows all four cultivated cotton species. There have been very few studies on the diversity and abundance of natural enemies of cotton insect pests in these cultivated cotton species. Therefore, the current study (2016–2018) was conducted to assess the diversity and abundance of natural enemies that cultivated cotton species harbour. Phule Dhanwantari, Suraj, Suvin, RCH-2, and DCH-32 were the five genotypes used in the study, each with a distinct genetic background. Using the *adiv* 2.0.1 and *vegan* R packages, we identified significant differences in natural enemies in terms of species diversity, richness, evenness, and abundance. Analysis of Similarity (ANOSIM) and Non-metric Multidimensional Scaling (NMDS) indicated substantial differences in the natural enemy community structure among the examined genotypes. A total of 17,279 natural enemies were collected and identified across genotypes from seven predatory families and five parasitoid families. The percentage share of these natural enemy families across genotypes and years, in descending order, is Coccinellidae (28.23%) < Tachinidae (19.23%) < Braconidae (12.68%) < Chrysopidae (11.65%) < Chalcididae (9.41%) < Aphelinidae (6.33%) < Pentatomidae (3.29%) < Ichneumonidae (2.37%) < Syrphidae (2.33%) < Vespidae (1.81%) < Asilidae (1.79%) < Geocoridae (0.89%). Coccinellidae, Tachinidae, Braconidae, Chrysopidae, Chalcididae, and Aphelinidae are the six major families that account for more than 85% of all recorded natural enemies. These six families have a higher percentage share in Phule Dhanwantary (90%) compared to the other genotypes. The conservation and better utilization of these natural enemies are crucial for the ecological and safe management of insect pests in the cotton ecosystem.

## Introduction

Cotton provides a livelihood to 6–6.5 million farmers and around 60 million people engaged in cotton-related activities in India^1^. The country is bestowed with diverse climatic conditions from the north to the south suitable to grow all four cultivated cotton species, viz*., Gossypium herbaceum, Gossypium arboreum, Gossypium barbadense*, and *Gossypium hirsutum*. The species *G. herbaceum* and *G. arboreum* are native to the old world, also called Asiatic cotton, whereas *G. hirsutum* and *G. barbadense* are the new world cotton species, called upland and Egyptian cotton, respectively. Because of its evolutionary history, *G. arboreum* is generally known as Desi cotton in India, where it has been farmed for over 5000 years. Before India’s independence (1947), it occupied 67% of the total cotton growing area^1^, but its acreage dropped dramatically (3% in 2021–22) with the introduction of upland cotton *G. hirsutum*, whereas *G. hirsutum* acreage rose prodigiously (> 94% in 2021–22)^2^. This paradigm shift favoured upland cotton, causing desi cotton to lose its lustre in its own country. The fundamental reason for the widespread adoption of upland cotton was the development of genetically modified (GM) cotton, specifically *G. hirsutum* with *Cry1Ac* (Bollgard) and stacked *Cry IAc* + *Cry2Ab* (Bollgard II) in 2002 and 2006, respectively. The GM cotton provided excellent control of bollworms, resulting in a significant reduction in the number and volume of insecticide sprays used for bollworm control^3^; however, the introduced GM technology has no controlling effect on sucking pests^4^, necessitating a greater number of insecticide sprays to keep their population under control^5^. Cotton insecticide use has increased, and indiscriminate insecticide use has various ecological repercussions, one of which is the disruption of pest-natural enemy interaction^6^. Several studies have been undertaken to evaluate the diversity and quantity of natural enemies in cotton. However, there is a significant paucity of studies comparing the abundance and diversity of insect natural enemies in the cultivated species of cotton viz*., G. arboreum, G. hirsutum, G. barbadense,* and interspecific cotton (*Gossypium hirsutum* X *Gossypium barbadense*). Therefore, the study was conducted with the primary goal of examining and comparing the diversity and abundance of insect natural enemies harboring in cultivated cotton species. The conservation and effective utilization of these natural enemies are of paramount importance for ecologically sound and safe insect pest management within the cotton ecosystem. This significance arises from the fact that the most commonly employed method for insect pest control in the cotton ecosystem is the application of insecticides.

## Results

### Species diversity and species richness

**Figure 1:**
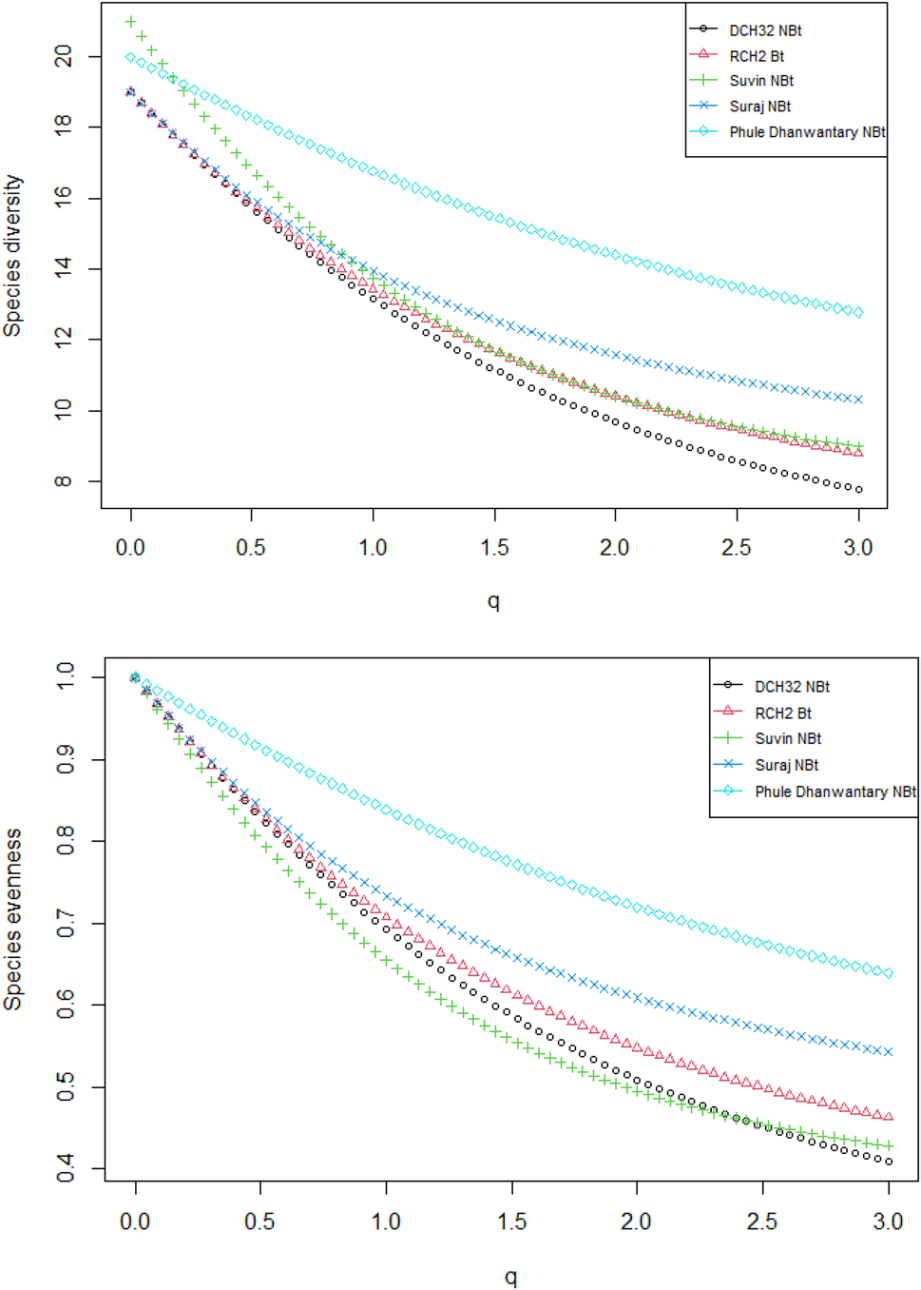
Species diversity and species evenness of natural enemies in the cotton test genotypes for the pooled data (2016–2018).

The species diversity of natural enemies differed greatly among the test genotypes. When abundance data are omitted (when, q = 0), the diversity indices profile of 2016 natural enemies data shows that the Phule Dhanwantary dominates in terms of species richness with 21 numbers (q = 0), followed by Suvin (18) and Suraj (12), with Suraj (12) having the lowest species richness (Fig. S1). When the importance of diversity to abundance is increased (q < 2), the diversity level of RCH 2 decreases marginally but stays higher than the rest of the genotypes. When the abundance data are discarded (when q = 0), the diversity indices profile of 2017 natural enemies data shows that the Phule Dhanwantary (17), Suvin (17), Suraj (17), and RCH2 (17) dominate in terms of species richness or number of species (q = 0), with DCH32 having the lowest species richness (Fig. S2). However, when the importance of diversity to abundance is increased (q < 1.5), the diversity level of RCH2 and Suvin is virtually as low as that of DCH32. When the importance of abundance is restored (q < 2), the diversity level of Suvin returns to a medium level, but Phule Dhanwantary and Suraj remain higher and DCH32 and RCH 2 remain low (Fig. S2). When abundance data are omitted (when q = 0), the diversity indices profile of 2018 natural enemies data shows that the Suvin (20) and Phule Dhanwantary (19) dominate in terms of species richness or a number of species, with RCH2 and Suraj having the lowest species richness (Fig. S3). However when diversity measures increase the importance of the abundance (*q* < 1.5) the diversity level of Suraj, RCH2 and DCH32 were almost equal and lower compare to Phule Dhanwantary and Suvin. When the importance of the abundance increases again (*q* = 2), the diversity level of RCH2 and Suraj recovers to the medium level while Phule dhanwantary remains higher and DCH32 remains lowest (Fig. S3). The diversity indices profile of pooled data (2016–2018) of natural enemies shows that when the abundance data are discarded (when *q* = 0), the Phule Dhanwantary (21) and Suvin (20) dominate in terms of species richness or a number of species (*q* = 0), with DCH32, RCH2 and Suraj having the lowest species richness (Fig. 1). However, when diversity measures increases the importance to the abundance (*q* < 0.5) the diversity level of Suraj, RCH2 and DCH32 were almost equal and lower compared to Phule Dhanwantary and Suvin. When the importance of the abundance increases again (*q* < 1.5), the diversity level of RCH2 and Suraj recovers to the medium level while Phule Dhanwantary remains higher and DCH32 remains lowest.

The diversity and species richness of insect natural enemies exhibited significant variations among the tested genotypes, as illustrated in Fig. 1 and Figs. S1 to S3 throughout the study duration. Notably, when comparing Phule Dhanwantary, a desi cotton variety, with RCH2, an introduced or New World cotton variety, desi cotton (Phule Dhanwantary) boasted higher levels of diversity and species richness compared to the introduced cotton (RCH2), as depicted in Fig. 1 and Figs. S1 to S3. However, there was no discernible demarcation in terms of the diversity and abundance of insect natural enemies between *Bt-*cotton (RCH2) and non-*Bt*-cotton varieties (Phule Dhanwantary, Suraj, Suvin, DCH32), as illustrated in Fig. 1 and Figs. S1 to S3.

### Species evenness

The evenness index indicates how evenly weighted species are in diversity measurement. The species' evenness indices range from 0 to 1, where evenness is maximum at 1 (when *q* = 0). When *q* increases the abundant species are overweighted more than the rare species, which results in the decrease of evenness of the species weight. The species evenness indices of 2016 natural enemies data showed the rate of decrease in species evenness varied with the genotypes (Fig. S1). The rate of decrease in species evenness with an increase in *q* was observed more in Phule Dhanwantary and Suvin compared to the rest of the test genotypes (Fig. S1), which indicates abundant species are overweighted compared to the rare species in these genotypes. During 2017 likewise, the rate of decrease in species evenness varied with the genotypes. The rate of decrease in species evenness with an increase in *q* was observed less in Phule Dhanwantary and Suraj compared to the rest of the test genotypes (Fig. S2), which indicates natural enemies species weights are even in these genotypes compared to the rest of the genotypes. Similarly, in 2018, the rate of decrease in species evenness varied with the genotypes. The rate of decrease in species evenness with an increase in *q* was observed less in Phule Dhanwantary and Suraj compared to the rest of the test genotypes (Fig. S3), which indicates natural enemies species weights are even in these genotypes compared to the rest of the genotypes. The species evenness indices profile of pooled data (2016–2018) showed that the rate of decrease in species evenness varied with the genotypes. The rate of decrease in species evenness with an increase in *q was* observed less in Phule Dhanwantary compared to the rest of the test genotypes (Fig. 1), which implies natural enemies species weights are even in Phule Dhanwantary compared to the rest of the genotypes.

Species richness, abundance, species diversity and evenness indices indicate the differences in natural enemies among the test genotypes. To complement these results the NMDS analysis was performed based on the similarity of natural enemies species composition using the Bray–Curtis index (stress = 0.01), which revealed the differences in the natural enemies community structure among test genotypes in 2016, 2017, 2018 and pooled data 2016–18 (Fig. S4). ANOSIM analysis was performed to complement the results obtained from NMDS analysis, the results revealed the significant differences in the natural enemies composition among the test genotypes in 2016 (*R* = 1, *P* = 0.0014), 2017 (*R* = 1, *P* = 0.0011), 2018 (*R* = 1, *P* = 0.0003) and in the pooled data (*R* = 1, *P* = 0.001), where *R*-value closes to 1.0 signify the dissimilarity between the groups (Cotton genotypes), whereas *R*-value closes to 0 signify the even distribution and *R*-value below 0 signify the dissimilarities are greater within the groups.

### Abundance

A total of 21 species of insect natural enemies of cotton insect pests were observed in the experimental plots during the study period of 2016–2018 (Table 1). Out of 21 recorded natural enemies, the abundance of *C. septempunctata *(*P < 0.05*)*, Geocoris sp. *(*P < 0.05*)*, Encarsia sp. *(*P < 0.05*)*, P. laxa *(*P < 0.05*)*, Polistes sp. *(*P < 0.05*) differed significantly among the test genotypes (Table 2, Figs. S5–S8), however, the abundance of all the natural enemies differed significantly among the sampling Standard Meteorological Weeks (SMW) (*P < 0.01*) and few natural enemies in the interaction effect of genotypes and sampling SMW (Table 2). The seasonal mean population density of natural enemies within each genotype (*P < 0.05*) differed significantly (Table S1).

**Table 1:** List of natural enemies collected and identified from the experimental plot during the study period.

Name of the natural enemies	Family	Order
Predators		
* Asilidae*	Asilidae	Diptera
* Brumoides suturalis* (Fabricius)	Coccinellidae	Coleoptera
* Cheilomenes sexmaculata* (Fabricius)	Coccinellidae	Coleoptera
* Chrysoperla zastrowi* (Esben-Petersen)	Chrysopidae	Neuroptera
* Coccinella septempunctata* Linnaeus	Coccinellidae	Coleoptera
* Eocanthecona furcellata* (Wolff)	Pentatomidae	Hemiptera
* Geocoris sp.*	Geocoridae	Hemiptera
* Ischiodon scutellaris* (Fab.)	Syrphidae	Diptera
* Nephus regularis* Sicard	Coccinellidae	Coleoptera
* Polistes sp.*	Vespidae	Hymenoptera
* Scymnus coccivora* Ayyar	Coccinellidae	Coleoptera
Parasitoids		
* Apanteles pectinophorae*	Braconidae	Hymenoptera
* Brachymeria sp.*	Chalcididae	Hymenoptera
* Bracon greeni* Ashmead	Braconidae	Hymenoptera
* Campoletis chlorideae* Uchida	Ichneumonidae	Hymenoptera
* Chelonus blackburni* (Cameron)	Braconidae	Hymenoptera
* Dirrhinus sp.*	Chalcididae	Hymenoptera
* Encarsia sp.*	Aphelinidae	Hymenoptera
* Eucelatoria bryani* Sabrosky	Tachinidae	Diptera
* Palexorista laxa* (Curran)	Tachinidae	Diptera
* Rhogas aligarhensis* Quadri	Braconidae	Hymenoptera

**Table 2:** F-values of mean seasonal insect natural enemies abundance during the study period 2016–2018.

Factors	DF	*C. septempunctata*	*C. sexmaculata*	*S. coccivora*	*B. suturalis*	*I. scutellaris*	*C. zastrowi*	*Geocoris sp.*	*Encarsia sp*	*C. blackburni*	*R. aligarhensis*	*E. bryani*
Genotype	410	4.85*	1.36 NS	1.09 NS	0.49 NS	0.41 NS	0.38 NS	4.68*	4.47*	0.83 NS	1.25 NS	0.72 NS
SMW	10,100	11.96**	7.92**	6.97**	18.35**	2.37*	7.38**	1.76 NS	7.94**	3.10**	6.08**	9.17**
Genotype* SMW	40,100	1.22 NS	3.35**	1.92**	5.01**	1.24 NS	0.74 NS	0.85 NS	3.33**	1.64*	1.62*	1.19 NS

Factors	DF	*C. chloridae*	*B. greeni*	*P. laxa*	*A. pectinophorae*	*N. regularis*	*E. furcellata*	*Brachymeria sp.*	*Dirrhinus sp.*	*Asilidae*	*Polistes sp.*	
Genotype	410	1.16 NS	3.18 NS	6.05**	1.5 NS	1.45 NS	0.98 NS	0.85 NS	1.13 NS	0.50 NS	5.80*	
SMW	10,100	13.10**	5.60**	5.58**	4.00**	2.22*	5.64**	5.39**	5.74**	4.59**	3.78**	
Genotype* SMW	40,100	1.21 NS	2.68**	1.97**	1.80**	1.01 NS	1.02 NS	1.75*	1.24 NS	2.43**	1.53*	

Repeated measures ANOVA, *P < 0.05, **P < 0.01, *NS* not statistically significant.

### Pest population dynamics in the test genotypes

The dynamics of the cotton insect pests during the entire study period were kept on watch from 33 to 43 SMW (Table 3). The most commonly encountered sucking pests in the test genotypes were aphid (*Aphis gossypii*), thrips (*Thrips palmi)*, whitefly (*Bemisia tabaci*), jassid (*Amrasca biguttula biguttula*) and mirid bugs (*Hyalopeplus lineifer, Campylomma livida and Creontiades biseratense*) and the lepidopteran bollworms viz*.,* American bollworm (*Helicoverpa armigera), Pink bollworm (Pectinophora gossypiella),* Spotted bollworm *(Earias vittella* Fab.) and Spiny bollworm (*E. insulana* Boisd.). The appearance of sucking pests began at 30 SMW and observed till 43 SMW, however, bollworms began to appear from 34 SMW and not beyond43 SMW. The incidence of sucking pests was not significantly differed among the test genotypes, however only aphids (*P < 0.01*) and whiteflies (*P < 0.01*) differed significantly among the sampling SMW across the years. However, there was no statistically significant difference in the interaction effect of genotypes and sampling SMW across the study years for all the pests (Table 4).

**Table 3:** The total counts of insect pests are observed in different cotton genotypes during the cotton growing season of 2016, 2017 and 2018.

Insect pests	2016					2017					2018				
	DCH32	RCH2	Suvin	Suraj	Phule	DCH32	RCH2	Suvin	Suraj	Phule	DCH32	RCH2	Suvin	Suraj	Phule
Aphid	3540	3403	4103	4461	2839	6870	5220	3660	5387	7622	15,952	22,410	6977	18,932	16,740
Whitefly	4035	4082	3460	4545	3760	5146	5108	4021	5376	4426	4687	3251	3542	3899	3067
Jassid	6026	5964	3538	5959	6890	7016	5697	3865	5730	7422	14,893	14,472	4460	9968	17,658
Thrips	3601	5582	3834	5161	4537	3967	3616	3119	4049	3344	6098	5875	4460	15,584	5659
Mirid bug	2228	2264	2224	2339	2416	2600	2357	2545	2558	2795	281	464	386	1069	97

**Table 4:** F-values of mean seasonal population density of insect pests of cotton during the study period 2016–2018.

Factors	DF	2016					2017					2018				
		Aphid	Whitefly	Jassid	Thrips	Mirid bug	Aphid	Whitefly	Jassid	Thrips	Mired bug	Aphid	Whitefly	Jassid	Thrips	Mirid bug
Genotype	4, 10	0.26 NS	1.34 NS	1.07 NS	1.07 NS	0.15 NS	0.31 NS	1.37 NS	1.25 NS	1.31 NS	0.22 NS	0.29 NS	1.41 NS	4.12**	1.11 NS	0.24 NS
SMW	10,100	3.88**	4.06**	0.53 NS	2.56**	1.36 NS	4.01**	4.36**	0.83 NS	3.35**	1.16 NS	3.08**	4.66**	0.52 NS	2.32**	1.38 NS
Genotype* SMW	40,100	0.64 NS	0.64 NS	1.01 NS	0.88 NS	0.62 NS	0.73 NS	0.61 NS	0.93 NS	1.07 NS	0.64 NS	0.39 NS	0.51 NS	1.08 NS	0.78 NS	0.64 NS

Repeated measures ANOVA, *P < 0.05, **P < 0.01, *NS* not statistically significant.

## Discussion

The 3-year data on the abundance and diversity of insect natural enemies of cotton insect pests demonstrated significant heterogeneity among the test genotypes. These selected test genotypes belong to different genetic backgrounds comprising diploid, tetraploid and an interspecific hybrid. *G. arboreum* (Phule Dhanwantari) is diploid, while *G. hirsutum* (Suraj and RCH-2) and *G. barbadense* (Suvin) are tetraploid and *G. hirsutum* X *G. barbadense* (DCH32) an interspecific hybrid. Only RCH2 was the GM cotton with *Cry1Ac* + *Cry2Ab* gene, whereas the rest were non-GM cotton genotypes. The study attempted to comprehend the diversity and abundance of natural enemies among genotypes from various genetic origins of cultivated cotton species. The diversity analysis revealed clear differences in natural enemies among the test genotypes. The diversity indices profile of natural enemies data indicated that Phule Dhanwantary and Suvin dominate in terms of species richness with DCH32, RCH2 and Suraj having the lowest species richness. The species richness, abundance, species diversity and evenness indices indicate the differences in natural enemies among the test genotypes. This was supported by the NMDS analysis based on the similarity of natural enemies species composition using the Bray–Curtis index (stress = 0.01) and it revealed the differences in the natural enemies community structure among test genotypes. The ANOSIM results corroborated the NMDS findings, indicating significant differences in natural enemy composition among test genotypes in each study year and for the pooled data.

When we compared the GM cotton genotype with non-GM cotton genotypes for the natural enemies' diversity and abundance, we found no significant changes, these results conform with the earlier reports^7–10^. However, few studies have demonstrated the adverse effects of *Bt* toxins on the natural enemies^7,11,12^, while others have reported the minor effects^13^. In contrast, equally ample reports are available to show the no negative effects of Bt toxins on insect natural enemies, no adverse effects of *Cry1Ab* on *Crysoperla carnea*^14,15^, and no adverse effect on natural enemy’s composition in transgenic *Cry1Ie* maize^16^ and other arthropods^15,17,18^. Besides, it is very important to understand the reason behind the reduction of the natural enemy’s population in the transgenic GM crop through long-term assessment projects because the reduction in the prey arthropod population in transgenic crop field might be the reason for the reduction in natural enemies population, Naranjo^19^ study revealed from the long-term assessment that the 19% reduction in major arthropods in *Bt* cotton fields linked to a reduction in the prey population.

All the natural enemies recorded in the experimental plot during the study period belonged to 12 different insect families, out of which seven were predator families viz*.,* Coccinellidae, Syrphidae, Chrysopidae, Pentatomidae, Vespidae Geocoridae and Asilidae and another five were parasitoid family viz*.,* Aphelinidae, Braconidae, Tachinidae, Ichneumonidae and Chalcididae (Table 1). A total of 17,279 natural enemies were recorded from all the genotypes from three years of the study period (Tables S2 and S3). The descending order of natural enemy families for the count data is Coccinellidae (4780) < Tachinidae (3403) < Braconidae (2176) < Chrysopidae (2094) < Chalcididae (1595) < Aphelinidae (1130) < Pentatomidae (567) < Ichneumonidae (407) < Syrphidae (367) < Vespidae (296) < Asilidae (311) < Geocoridae (153). The per cent share of the natural enemy families across genotypes and years in the descending order is Coccinellidae (28.23%) < Tachinidae (19.23%) < Braconidae (12.68%) < Chrysopidae (11.65%) < Chalcididae (9.41%) < Aphelinidae (6.33%) < Pentatomidae (3.29%) < Ichneumonidae (2.37%) < Syrphidae (2.33%) < Vespidae (1.81%) < Asilidae (1.79%) < Geocoridae (0.89%) (Table S3). More than 85% per cent of the total natural enemies recorded belong to the six major families viz*.,* Coccinellidae, Tachinidae, Braconidae, Chrysopidae, Chalcididae and Aphelinidae. While, the natural enemies of the families such as Syrphidae, Vespidae, Asilidae and Geocoridae were least recorded (Table S3). The increased and decreased abundance of natural enemies might be due to the availability of suitable prey on the crop (Naranjo^19^). The per cent share of natural enemies belonging to the major six families is more in Phule Dhanwantary (≈ 90%) compare to the rest of the genotypes. The incidence of insect pests was found significantly different among the test genotypes (Table 4). The incidence of sucking pest and bollworms were found less in Phule Dhanwantary compared to the other non-GM cotton genotypes, as the genotype proved tolerant to many cotton insect pests in India^20^. The incidence of bollworms was found negligible to nil in RCH2, due to the presence of *Bt-Cry* genes in them, however, there was a considerable incidence of sucking pests because *Bt-Cry* genes do not affect sucking pests. However, the incidence of *Thrips palmi* and mirid bugs were found non-significant across the years and genotypes.

From various other statistical analyses, it revealed that the species diversity, abundance, species richness and species evenness are significantly different among the test genotypes. The Phule Dhanwantary, a desi cotton genotype more tolerant to the insect’s pests of cotton harboured significantly more species of natural enemies compared to the rest of the cotton genotypes. In addition, the varieties of desi cotton, *G. arboreum* with high micronaire, high fluid absorbency and low ash content are ideal for surgical/absorbent use^21^. The revival of desi cotton is very essential because of its many important roles viz*.,* tolerance to biotic and abiotic stresses and its role in the surgical industry. Thus, it is imperative to explore *G. arboreum* genotypes further for the management of cotton insect pests through resistance breeding programmes.

## Conclusion

*Cheilomenes sexmaculata, Coccinella septempunctata, Brumoides suturalis, Chrysoperla zastrowi, Palexorista laxa and Dirrhinus sp*. of insect natural enemies were the most commonly and abundantly found insect natural enemies in the selected genotypes of cultivated species of cotton. The conservation and better utilization of these natural enemies are very crucial for the ecological and safe management of insect pests in the cotton ecosystem, where the most common practice followed for the management of insect pests is the insecticide application. The conservation and better utilization of these natural enemies might reduce the application of insecticide and various ecological repercussions which arises from the indiscriminate use of insecticides.

## Materials and methods

The field experiment was conducted at ICAR-Central Institute for Cotton Research (CICR), an experimental farm area in Nagpur, India (21°02′ 25.5″ N 79° 03′ 36.6″ E. The experiment was carried out under rainfed conditions for three years in a row, from 2016 to 2018. During the study period, the experimental site’s average lowest and maximum temperatures and rainfall were 17.33, 38.45 °C, and 0 327 mm, respectively.

### Cotton species and genotypes

*G. arboretum*, *G. hirsutum*, *G. barbadense*, and an interspecific hybrid (*G. hirsutum* X *G. barbadense*) were the cultivated cotton species employed in the experiment. Phule Dhanwantari (*G. arboretum*)*,* Suraj (*G. hirsutum*)*,* Suvin (*G. barbadense*), RCH-2 (*G. hirsutum*), and DCH-32 or Jayalaxmi (*Gossypium hirsutum* X *Gossypium barbadense*) were the five genotypes employed in the study. Among these only RCH-2 was *Bacillus thuringiensis* (Bt) cotton hybrid with *Cry IAc* + *Cry2Ab* genes.

### Experimental site

All five genotypes were grown separately in a 15 m × 42 m (0.157 acre) plot side by side with a gap of 3 m distance between them. As arthropods move more frequently on edges^22^, to exclude the edge effect, there followed a 3 m distance between each genotype and avoided sampling in the edge area, thus only central rows of plots were sampled. From sowing till harvest the CICR recommended agronomic practices were followed. During the entire study period, no pesticides were applied to the crop and the experiment was repeated three times.

### Dynamics and diversity of natural enemies

The abundance and diversity of natural enemies were assessed by the yellow pan trap sampling method. The sampling was done by setting up of yellow pan trap (YPT) at the rate of 20 traps per ha. The YPTs were 20 cm in diameter and 8 cm in height, which were kept randomly by filling 3/4th of water in it, addition added a pinch of colourless detergent to reduce the density of the water to make the trapped insect settle at the bottom and also added a pinch of salt to reduce the disintegration of the tissues of the insect which are trapped in. After 24 h, the trapped insects were collected and processed for identification by storing them in 70% ethanol. The sample collections were recorded at the weekly interval, starting from the seedling stage to the harvest stage of the crop. Identification of the collected insects was done by the taxonomy section of the Entomology Division, IARI, New Delhi, India.

### Population dynamics of insect pests

The dynamics of the cotton insect pests during the entire study period were recorded from 33 to 43 SMW.

### Statistical analysis

The natural enemy count data was analysed in R software^23^ using the adiv 2.0.1^24^ and vegan 2.5–7^25^ packages to assess the abundance, species richness, evenness, and diversity of natural enemies in the test genotypes. Diversity indices viz*.,* Shannon^26^, Simpson^27^, GiniSimpson^28^, Margalef^29^, Menhinick^30^ and McIntosh^31^ were used for diversity analysis and indices viz*.,* Shannon, Simpson, GiniSimpson, Heip^32^, McIntosh and Smith Wilson^33^ were used for species evenness analysis by using *adiv* 2.0.1 package. Hill numbers (or the effective number of species) were used to quantify species/taxonomic diversity; they are an intuitive and statistically superior alternative to conventional diversity indices. The sensitivity of the measurements to species relative abundances is determined by a diversity order q, which is parameterized by hill numbers. Hill numbers include the three most widely used species diversity measures as special cases: species richness (q = 0), Shannon diversity (q = 1) and Simpson diversity (q = 2). *Non-metric multidimensional scaling (NMDS) analysis* was performed by using a *vegan* 2.5–7 package of R^25^. The NMDS analysis based on the similarity of natural enemies species composition was evaluated using the Bray–Curtis index^34^ to assess the differences in the natural enemy’s community structure in the tests genotypes. *The analysis of similarity (ANOSIM)* was performed in PAST4.03 software^35^ to test the significant differences in natural enemies composition among the cotton genotypes. The ANOSIM examined if rank similarities within groups (sample points of cotton genotype) are greater than those between groups (Cotton genotypes) using 10,000 random reassignments of observed species data^36^. The two-way repeated measures Analysis of Variance (ANOVA) was used to assess the difference in insect natural enemy abundance among genotypes (between-subject factor) and sampling standard meteorological weeks (within-subject factor), where the sampling date, i.e. standard meteorological weeks, was repeated within replicates. Tukey’s HSD post hoc test was used to determine whether there are any statistically significant differences in the abundance of natural enemies in each genotype using repeated measures one-way ANOVA. Repeated measures two-way ANOVA was used similarly to investigate if there are any significant differences in the mean seasonal population of cotton insect pests, here also the sampling date, i.e. standard meteorological weeks was repeated within replicates.

### Plant guidelines

The use of plant parts in the study complies with international, national, and/or institutional guidelines.

### Supplementary Information

Supplementary Information.

## Data Availability

The datasets generated during and/or analysed during the current study are available from the corresponding author on reasonable request. All data generated or analysed during this study are included in this article (and its supplementary information files).

## References

[1] DCD. Status paper of Indian cotton. *Directorate of Cotton Development (DCD), Ministry of Agriculture and Farmers Welfare, Department of Agriculture, Cooperation and Farmers Welfare (DAC & FW), Government of India*, 1–211, https://www.nfsm.gov.in/StatusPaper/Cotton2016.pdf (2017).

[2] ICAC Data portal; Production of cotton lint in ‘000 metric tonnes. *International Cotton Advisory Committee (ICAC). Washington, USA* (2019/20). https://www.icac.org/DataPortal/DataPortal?Year=2021/22%20proj.

[3] Krishna VV, Qaim M (2012). Bt cotton and sustainability of pesticide reductions in India. Agric. Syst..

[4] Sharma HC, Pampapathy G (2006). Influence of transgenic cotton on the relative abundance and damage by target and non-target insect pests under different protection regimes in India. J. Crop Prot..

[5] Kranthi KR, Stone GD (2020). Long-term impacts of *Bt* cotton in India. Nat. Plants.

[6] He Y, Zhao J, Zheng Y (2012). Lethal effect of imidacloprid on the coccinellid predator *Serangium japonicum* and sublethal effects on predator voracity and on functional response to the whitefly *Bemisia tabaci*. Ecotoxicology.

[7] Head G, Moar M, Eubanks M, Freeman B, Ruberson J, Hagerty A, Turnipseed S (2005). A multiyear, large-scale comparison of arthropod populations on commercially managed Bt and non-Bt cotton fields. Environ. Entomol..

[8] Torres JB, Ruberson JR (2005). Canopy- and ground-dwelling predatory arthropods in commercial Bt and non-Bt cotton fields: Patterns and mechanisms. Environ. Entomol..

[9] Sharma HC, Arora R, Pampapathy G (2007). Influence of transgenic cottons with *Bacillus thuringiensis* cry1Ac gene on the natural enemies of *Helicoverpa armigera*. Biocontrol.

[10] Wei-Di L, Kong-Ming W, Xue-Xin C, Hong-Qiang F, Guang X, Yu-Yuan G (2004). Effect of transgenic cotton carrying CryA + CpTI + Cry1Ac genes on diversity of arthropod communities in cotton field in North China. Chin. J. Agric. Biotechnol..

[11] Dutton A, Klein H, Romeis J, Bigler F (2003). Prey-mediated effects of *Bacillus thuringiensis* spray on the predator *Chrysoperla carnea* in maize. Biol. Control.

[12] Pilcher CD, Rice ME, Obrycki JJ (2005). Impact of transgenic *Bacillus thuringiensis* corn and crop phenology on five nontarget arthropods. Environ. Entomol..

[13] Sisterson MS, Biggs W, Olson C, Carrière Y, Dennehy TJ, Tabashnik BE (2004). Arthropod abundance and diversity in Bt and non-Bt cotton fields. Environ. Entomol..

[14] Romeis J, Dutton A, Bigler F (2004). *Bacillus thuringiensis* toxin (CryA1b) has no direct effect on the green lacewing *Chrysoperla carnea* (Stephens) (Neuroptera; Chrysopidae). J. Insect. Physiol..

[15] Dhillon MK, Pampapathy G, Wadaskar RM, Sharma HC (2012). Impact of *Bt* transgenic cottons and insecticides on target and non-target insect pests, natural enemies and seed cotton yield in India. Indian J. Agric. Sci..

[16] Guo J, He K, Hellmich RL, Bai B, Zhang T, Liu Y, Ahmed T, Wang Z (2016). Field trials to evaluate the effects of transgenic cry1Ie maize on the community characteristics of arthropod natural enemies. Sci. Rep..

[17] Phulse VB, Udikeri SS (2014). Seasonal incidence of sucking insect pests and predatory arthropods in desi and Bt transgenic cotton. Karnataka J. Agric. Sci..

[18] Zhao C-Y, Yu X-D, Liu Y-B, Li J-S (2016). Effects of insect-resistant transgenic cotton on ground-dwelling beetle assemblages (Coleoptera). J. Integr. Agric..

[19] Naranjo SE (2005). Long-term assessment of the effects of transgenic Bt cotton on the function of the natural enemy community. Environ. Entomol..

[20] Vonzun S, Messmer MM, Boller T, Shrivas Y, Patil SS, Riar A (2019). Extent of bollworm and sucking pest damage on modern and traditional cotton species and potential for breeding in organic cotton. Sustainability.

[21] Nachane RP, Nagarkar RD, Mehetre SS, Patil VR, Mokate AS, Shinde GC (2004). Studies on efficacy of single-stage process and suitability of two *G*. *aboreum* cottons for production of absorbent cotton. J. Indian Soc. Cotton Improv..

[22] Allema, B. Quantifying and simulating movement of the predator carabid beetle *Pterostichus melanarius* in arable land. Wageningen University and Research. https://edepot.wur.nl/301867 (2014).

[23] R Core Team R: A language and environment for statistical computing. R Foundation for Statistical Computing, Vienna, Austria. https://www.R-project.org/ (2021).

[24] Pavoine S (2020). adiv: An r package to analyse biodiversity in ecology. Methods Ecol. Evol..

[25] Oksanen, J., Blanchet, F.G., Friendly, M., Kindt, R., Legendre, P., McGlinn, D., Wagner, H. *vegan: Community Ecology Package*. R package version 2.5–6. https://CRAN.R-project.org/package=vegan (2019).

[26] Shannon CE (1948). A mathematica theory of communication. Bell. Syst. Tech. J..

[27] Simpson EH (1949). Measurement of diversity. Nature.

[28] Gini C (1912). Variabilita e Mutabilita.

[29] Margalef R (1972). Homage to Evelyn Hutchinson, or why there is an upper limit to diversity. Conn. Acad. Arts Sci..

[30] Menhinick EF (1964). A comparison of some species-individuals diversity indices applied to samples of field insects. Ecology.

[31] McIntosh RP (1967). An index of diversity and the relation of certain concepts to diversity. Ecology.

[32] Heip C (1974). A new index measuring evenness. J. Mar. Biol. Assoc. U.K..

[33] Smith B, Wilson JB (1996). A consumer’s guide to evenness indices. Oikos.

[34] Bray JR, Curtis JT (1957). An ordination of upland forest communities of southern Wisconsin. Ecol. Monogr..

[35] Hammer Ø, Harper DAT, Ryan PD (2001). PAST: Paleontological statistics software package for education and data analysis. Palaeontol. Electron..

[36] Clarke KR (1993). Non-parametric multivariate analyses of changes in community structure. Aust. J. Ecol..

