# Supplementary material for: The comparison of species diversity and abundance of insect natural enemies in the domesticated species of cotton using the yellow pan trap method
Source: Sci Rep. 2024 Feb 2;14:2787. doi: 10.1038/s41598-023-48347-3 (PMC10837446; doi:10.1038/s41598-023-48347-3)
Supplement: Supplementary file 1 — Supplementary Information. [file 41598_2023_48347_MOESM1_ESM.docx]

**Table S1: Seasonal mean population density (Mean± SEM)) of natural enemies during 2016, 2017 and 2018 at the experimental plot of ICAR-CICR, Nagpur**

| **Insect**  **natural enemies** | **2016** | | | | | | **2017** | | | | | | **2018** | | | | |
| --- | --- | --- | --- | --- | --- | --- | --- | --- | --- | --- | --- | --- | --- | --- | --- | --- | --- |
|  | **DCH32** | **RCH2** | **Suvin** | **Suraj** | **Phule** | **DCH32** | | **RCH2** | **Suvin** | **Suraj** | **Phule** | **DCH32** | | **RCH2** | **Suvin** | **Suraj** | **Phule** |
| ***A. pectinophorae*** | 1.2  ±0.28jihg | 1.26  ±0.24ghi | 1.82  ±0.08fgh | 1.22  ±0.08efg | 1.22  ±0.36fghi | | 1.802  ±0.28gh | 1.27  ±0.28gh | 1.16  ±0.29fg | 2.88  ±0.75ghi | 1.82  ±0.22fg | | 1.46  ±0.71e | 1.1  ±0.22gh | 2.36 ±0.43e | 1.02  ±0.37h | 1.7  ±0.29ghij |
| ***Asilidae*** | 1.92  ±0.37fgh | 1.72  ±0.16fghi | 1.84  ±0.08fgh | 0.00 ±0.00h | 0.64  ±0.16ghij | | 1.242  ±0.29gh | 1.14  ±0.14gh | 1.76  ±0.29efg | 1.9  ±0.29ijkl | 1.1  ±0.80g | | 1.64  ±0.54e | 1.1  ±0.80gh | 2.36  ±0.43e | 1.2  ±1.13h | 1  ±0.59ij |
| ***B. greeni*** | 4.82  ±0.8cd | 3.58  ±0.36de | 4.78  ±0.36d | 1.16  ±0.16efg | 3.06  ±0.24de | | 4.104  ±0.67ef | 1.15  ±0.14gh | 5.3  ±0.22bc | 2.3  ±0.29hijk | 6.08  ±1.07cd | | 5.48  ±0.22c | 4.7  ±0.22d | 4.74  ±0.65d | 1.08  ±0.37h | 3.62  ±0.36cde |
| ***B. suturalis*** | 3.56 ±0.29de | 3.04  ±0.43ef | 3.06  ±0.14defg | 4.24  ±0.37bc | 18.8  ±1.70a | | 6.18  ±1.12cd | 4.08  ±0.28d | 4.72  ±0.59bcd | 4.26  ±0.79de | 4.2  ±0.28de | | 3.66  ±0.37d | 4.76  ±0.43d | 4.18  ±0.36d | 2.4  ±0.28fg | 3.06  ±0.14defg |
| ***Brachymeria sp.*** | 1.82  ±0.36fghi | 4.87  ±1.00cd | 4.32  ±2.14de | 2.96  ±0.29d | 1.34  ±1.24fghj | | 2.284  ±0.75g | 7.24  ±1.84c | 6.7  ±1.36b | 4.16  ±0.57def | 4.24  ±0.43de | | 1.66  ±0.42e | 6.1  ±1.5c | 1.14  ±0.14 | 4.2  ±0.28bc | 1.12  ±0.16ij |
| ***C. blackburni*** | 2.38 ±0.22efg | 1.24  ±0.29ghi | 1.26  ±0.14gh | 1.78  ±0.22ef | 1.78  ±0.22fgh | | 1.82  ±0.28gh | 1.24  ±0.36gh | 1.26  ±0.14fg | 0.46  ±0.94m | 0.66  ±0.14g | | 1.16  ±0.57e | 1.17  ±0.24gh | 1.42  ±0.85 | 2.76  ±0.57h | 0.5  ±1.08j |
| ***C. chloridae*** | 1.82  ±0.8fghi | 1.26  ±0.14ghi | 1.78  ±0.22fgh | 1.12  ±0.45fg | 1.8  ±0.14fgh | | 4.362  ±1.18e | 1.33  ±0.14gh | 1.54  ±1.31fg | 2.34  ±0.51hijk | 1.2  ±0.28g | | 1.7  ±0.54e | 2.32  ±0.45ef | 2.34  ±0.14e | 1.12  ±0.45h | 1.22  ±0.36hij |
| ***C. septempunctata*** | 1.3 ±0.28jihg | 1.73  ±0.22fgh | 3.1 ±0.28defg | 1.67  ±0.14ef | 2.32  ±0.5ef | | 1.68  0±0.22 | 1.78  ±0.78fgh | 2.22  ±0.08ef | 3.98  ±0.59efg | 3.7  ±0.29ef | | 1.26  ±0.37e | 1.75  ±0.14gh | 4.13  ±0.36d | 3.43  ±0.28cd | 1.27  ±0.14hij |
| ***C. sexmaculata*** | 6.29±1.56b | 5.46  ±3.93bc | 9.5  ±3.8b | 7.67  ±2.06a | 13.92  ±2.7b | | 6.65  0±1.77c | 7.68  ±2.14c | 6.7  ±1.63b | 6.14  ±1.24bc | 7.02  ±0.51bc | | 9.6  ±0.28b | 6.06  ±0.37c | 7.16  ±0.57c | 4.8  ±1.56b | 11.87  ±3.93a |
| ***C. zastrowi sillemi*** | 7.1 ±0.57b | 7.33  ±1.51a | 8.52  ±2.02b | 3.6  ±0.50cd | 10.26  ±2.07c | | 14.88  ±3.67b | 15.5  ±1.86b | 11.4  ±4.53a | 5.1  ±2.30cd | 12.02  ±5.31a | | 4.88  ±1.42cd | 7.96  ±1.59b | 12.9  ±3.38b | 3.64  ±0.29cd | 3.68  ±0.59cde |
| ***Dirrhinus sp.*** | 3.58  ±0.21de | 2.46  ±0.14efg | 1.16  ±0.57gh | 1.82  ±0.08ef | 1.8  ±0.28fgh | | 6.688  ±1.88c | 2.98  ±0.5def | 5.52  ±1.30bc | 1.22  ±0.22jklm | 1.24  ±0.29g | | 3.54  ±1.07d | 5.3  ±0.29cd | 7.78  ±0.64c | 1.68  ±0.62gh | 5.34  ±0.51c |
| ***E. bryani*** | 0.00 ±0.00i | 0.8  ±1.63hi | 1.76  ±0.29fgh | 1.24  ±0.08efg | 1.28  ±0.16fghj | | 4.742  ±0.65de | 2.44  ±0.29defg | 1.14  ±0.14fg | 3.08  ±0.22fgh | 5.38  ±0.22cde | | 1.1  ±0.41e | 2.46  ±0.14ef | 1.88  ±0.45 | 2.36  ±0.31fg | 1.12  ±0.45ij |
| ***E. furcellata*** | 0.5  ±0.41ji | 0.6  ±1.41hi | 1.24  ±1.56gh | 1.2  ±0.28efg | 1.84  ±0.08fg | | 2.09  ±1.48g | 1.91  ±0.54fg | 5.24  ±2.71bc | 2.36  ±0.57hij | 1.8  ±0.57g | | 3.5  ±2.77d | 1.02  ±0.62gh | 5.38  ±0.36d | 4.74  ±0.14b | 4.22  ±0.36cd |
| ***Encarsia sp*** | 3.5 ±0.28de | 2.5  ±0.28efg | 5.14  ±3.24c | 3.6  ±0.28cd | 1.22  ±0.8fghi | | 4.18  ±0.36ef | 3.65  ±0.43de | 12.3  ±1.93a | 6.48  ±0.88b | 4.8  ±0.28de | | 4.8  ±0.28cd | 1.74  ±0.51fg | 8.2  ±3.36c | 4.2  ±0.28bc | 2.34  ±0.51efghi |
| ***Geocoris sp.*** | 0.00 ±0.00i | 0.61  ±0.8hi | 1.18  ±0.22gh | 0.00 ±0.00h | 0.00 ±0.00i | | 0.00 ±0.00h | 0.2  ±0.82h | 0.4  ±1.63g | 1.32  ±1.3jklm | 1.88  ±1.2fg | | 1.12  ±0.45e | 0.44  ±0.91h | 1.14  ±0.14 | 0.00 ±.00i | 1.8  ±3.94fghij |
| ***I. scutellaris*** | 3.1 ±0.28ef | 0.19  ±0.82i | 1.82  ±0.08fgh | 2.99  ±0.28d | 0.59  ±0.15hij | | 1.62  ±0.99gh | 1.22  ±0.36gh | 0.2  ±0.82g | 1.04  ±0.65lm | 1.82  ±0.08fg | | 1.06  ±0.7e | 0.2  ±0.82h | 1.74  ±.79e | 3.4  ±0.94de | 3.58  ±1.63cdef |
| ***N. regularis*** | 1.78  ±0.22fghi | 1.14  ±0.14ghi | 1.2  ±0.28gh | 1.84  ±0.08e | 1.16  ±0.16fghij | | 1.746  ±0.14gh | 1.24  ±1.28gh | 3.56  ±0.43cde | 0.6  ±0.71m | 4.18  ±0.36e | | 1.78  ±0.36e | 2.92  ±0.59e | 2.4  ±0.28e | 1.28  ±0.45h | 2.94  ±0.51defgh |
| ***P. laxa*** | 17.1  ±0.36a | 6.9  ±2.0ab | 15.8  ±4.83a | 4.76  ±0.16b | 4.14  ±0.37d | | 19.18  ±3.04a | 21  ±4.12a | 12  ±1.77a | 8.84  ±1.59a | 8.16  ±3.15b | | 21.8  ±3.08a | 12.52  ±1.42a | 24.2  ±3.22a | 8.98  ±0.36a | 9.34  ±1.19b |
| ***Polistes wasp*** | 0.64  ±0.16jih | 1.24  ±0.8ghi | 0.72  ±0.37h | 0.8  ±0.82g | 0.4  ±0.82ij | | 0.624  ±1.41gh | 1.72  ±0.59fgh | 1.28  ±1.28fg | 1.18  ±0.22klm | 1.76  ±0.29g | | 1.2  ±0.42e | 1  ±0.59gh | 2.38  ±0.36e | 3.54  ±0.37cde | 1.18  ±0.22hij |
| ***R.aligarhensis*** | 5.4  ±0.29c | 2.42  ±0.08efg | 3.62  ±0.36def | 1.8  ±0.28ef | 1.76  ±0.29fgh | | 2.38  ±0.22fg | 2.39  ±0.45efg | 4.82  ±0.36bcd | 2.36  ±0.08hij | 4.86  ±0.14de | | 3.62  ±0.36d | 2.392  ±0.45ef | 4.84  ±0.57d | 1.12  ±0.86h | 1.22  ±0.36hij |
| ***S. coccivora*** | 3.02 ±0.22ef | 2.34  ±0.43efg | 2.46  ±0.37efgh | 1.24  ±0.29efg | 4.2  ±0.28d | | 4.82  ±0.42de | 2.99  ±0.22def | 3.06  ±0.14def | 3.58  ±0.64efg | 4.24  ±0.08de | | 1.84  ±0.57e | 4.66  ±2.07d | 4.26  ±0.37d | 3.56  ±0.43cd | 3.58  ±0.36cdef |

***** Means with the same letter in each genotype in each sampling year are not significantly different (α = 0.05)

**Table S2. The total counts of insect natural enemies observed in different cotton genotypes during the growing season of 2016, 2017 and 2018**

| **Insect**  **natural enemies** | **Total counts of natural enemies** | | | | | | | | | | | | | | |
| --- | --- | --- | --- | --- | --- | --- | --- | --- | --- | --- | --- | --- | --- | --- | --- |
|  | **2016** | | | | | **2017** | | | | | **2018** | | | | |
|  | **DCH32** | **RCH2** | **Suvin** | **Suraj** | **Phule** | **DCH32** | **RCH2** | **Suvin** | **Suraj** | **Phule** | **DCH32** | **RCH2** | **Suvin** | **Suraj** | **Phule** |
| *A. pectinophorae* | 18 | 19 | 27 | 18 | 18 | 27 | 18 | 17 | 43 | 27 | 25 | 17 | 35 | 15 | 26 |
| *B. greeni* | 72 | 54 | 72 | 17 | 46 | 62 | 17 | 80 | 35 | 91 | 82 | 71 | 71 | 16 | 54 |
| *B. suturalis* | 53 | 46 | 46 | 64 | 297 | 93 | 61 | 71 | 64 | 63 | 55 | 71 | 63 | 36 | 46 |
| *Brachymeria sp.* | 27 | 73 | 65 | 44 | 20 | 34 | 109 | 101 | 62 | 64 | 25 | 92 | 17 | 63 | 17 |
| *C. blackburni* | 36 | 19 | 19 | 27 | 27 | 27 | 18 | 19 | 7 | 10 | 17 | 18 | 21 | 41 | 8 |
| *C. chloridae* | 27 | 19 | 27 | 17 | 27 | 65 | 18 | 23 | 35 | 18 | 26 | 35 | 35 | 17 | 18 |
| *C. septempunctata* | 18 | 27 | 45 | 27 | 35 | 27 | 27 | 36 | 55 | 56 | 19 | 28 | 63 | 54 | 19 |
| *C. sexmaculata* | 108 | 85 | 293 | 116 | 209 | 100 | 115 | 100 | 92 | 105 | 144 | 91 | 107 | 72 | 180 |
| *C. zastrowi sillemi* | 118 | 110 | 248 | 54 | 187 | 223 | 233 | 170 | 75 | 180 | 73 | 119 | 194 | 55 | 55 |
| *Dirrhinus sp.* | 54 | 37 | 17 | 27 | 27 | 100 | 45 | 83 | 18 | 19 | 53 | 80 | 117 | 25 | 80 |
| *E. bryani* | 0 | 12 | 26 | 19 | 19 | 71 | 37 | 17 | 46 | 81 | 17 | 37 | 28 | 35 | 17 |
| *E. furcellata* | 8 | 9 | 19 | 18 | 28 | 32 | 29 | 79 | 35 | 27 | 53 | 15 | 81 | 71 | 63 |
| *Encarsia sp* | 54 | 36 | 178 | 54 | 18 | 63 | 55 | 184 | 97 | 72 | 72 | 26 | 123 | 63 | 35 |
| *Geocoris sp.* | 0 | 10 | 18 | 0 | 0 | 0 | 3 | 6 | 20 | 28 | 17 | 7 | 17 | 0 | 27 |
| *I. scutellaris* | 45 | 3 | 27 | 45 | 9 | 24 | 18 | 3 | 16 | 27 | 16 | 3 | 26 | 51 | 54 |
| *N. regularis* | 27 | 17 | 18 | 28 | 17 | 26 | 19 | 53 | 9 | 63 | 27 | 44 | 36 | 19 | 44 |
| *P. laxa* | 275 | 104 | 237 | 71 | 62 | 288 | 316 | 180 | 133 | 122 | 327 | 188 | 363 | 135 | 140 |
| *Polistes wasp* | 10 | 19 | 11 | 12 | 6 | 9 | 26 | 19 | 18 | 26 | 18 | 15 | 36 | 53 | 18 |
| *R.aligarhensis* | 80 | 36 | 54 | 27 | 26 | 36 | 35 | 72 | 35 | 73 | 54 | 36 | 73 | 17 | 18 |
| *Robberfly* | 29 | 26 | 28 | 0 | 10 | 19 | 17 | 26 | 29 | 17 | 25 | 17 | 35 | 18 | 15 |
| *S. coccivora* | 45 | 37 | 37 | 19 | 63 | 72 | 45 | 46 | 54 | 64 | 28 | 70 | 64 | 53 | 54 |

**Table S3. The Total counts of insect natural enemy family are observed and their per cent share in different cotton genotypes during the growing season of 2016, 2017 and 2018**

| **Family of insect**  **natural enemies** | **2016** | | | | | **2017** | | | | | **2018** | | | | |
| --- | --- | --- | --- | --- | --- | --- | --- | --- | --- | --- | --- | --- | --- | --- | --- |
|  | **DCH32** | **RCH2** | **Suvin** | **Suraj** | **Phule** | **DCH32** | **RCH2** | **Suvin** | **Suraj** | **Phule** | **DCH32** | **RCH2** | **Suvin** | **Suraj** | **Phule** |
| Aphelinidae | 54 | 36 | 178 | 54 | 18 | 63 | 55 | 184 | 97 | 72 | 72 | 26 | 123 | 63 | 35 |
| Asilidae | 29 | 26 | 28 | 0 | 10 | 19 | 17 | 26 | 29 | 17 | 25 | 17 | 35 | 18 | 15 |
| Braconidae | 206 | 128 | 172 | 89 | 117 | 152 | 88 | 188 | 120 | 201 | 178 | 142 | 200 | 89 | 106 |
| Chalcididae | 81 | 110 | 82 | 71 | 47 | 134 | 154 | 184 | 80 | 83 | 78 | 172 | 134 | 88 | 97 |
| Chrysopidae | 118 | 110 | 248 | 54 | 187 | 223 | 233 | 170 | 75 | 180 | 73 | 119 | 194 | 55 | 55 |
| Coccinellidae | 251 | 212 | 439 | 254 | 621 | 318 | 267 | 306 | 274 | 351 | 273 | 304 | 333 | 234 | 343 |
| Geocoridae | 0 | 10 | 18 | 0 | 0 | 0 | 3 | 6 | 20 | 28 | 17 | 7 | 17 | 0 | 27 |
| Ichneumonidae | 27 | 19 | 27 | 17 | 27 | 65 | 18 | 23 | 35 | 18 | 26 | 35 | 35 | 17 | 18 |
| Pentatomidae | 8 | 9 | 19 | 18 | 28 | 32 | 29 | 79 | 35 | 27 | 53 | 15 | 81 | 71 | 63 |
| Syrphidae | 45 | 3 | 27 | 45 | 9 | 24 | 18 | 3 | 16 | 27 | 16 | 3 | 26 | 51 | 54 |
| Tachinidae | 275 | 116 | 263 | 90 | 81 | 359 | 353 | 197 | 179 | 203 | 344 | 225 | 391 | 170 | 157 |
| Vespidae | 10 | 19 | 11 | 12 | 6 | 9 | 26 | 19 | 18 | 26 | 18 | 15 | 36 | 53 | 18 |
| **Family of insect**  **natural enemies** | **Per cent share of insect natural enemies family’s (%)** | | | | | | | | | | | | | | |
|  | **2016** | | | | | **2017** | | | | | **2018** | | | | |
|  | **DCH32** | **RCH2** | **Suvin** | **Suraj** | **Phule** | **DCH32** | **RCH2** | **Suvin** | **Suraj** | **Phule** | **DCH32** | **RCH2** | **Suvin** | **Suraj** | **Phule** |
| Aphelinidae | 4.89 | 4.51 | 11.77 | 7.67 | 1.56 | 4.51 | 4.36 | 13.29 | 9.92 | 5.84 | 6.14 | 2.41 | 7.66 | 6.93 | 3.54 |
| Asilidae | 2.63 | 3.26 | 1.85 | 0.00 | 0.87 | 1.36 | 1.35 | 1.88 | 2.97 | 1.38 | 2.13 | 1.57 | 2.18 | 1.98 | 1.52 |
| Braconidae | 18.66 | 16.04 | 11.38 | 12.64 | 10.17 | 10.87 | 6.98 | 13.57 | 12.27 | 16.30 | 15.17 | 13.15 | 12.46 | 9.79 | 10.73 |
| Chalcididae | 7.34 | 13.78 | 5.42 | 10.09 | 4.08 | 9.59 | 12.21 | 13.29 | 8.18 | 6.73 | 6.65 | 15.93 | 8.35 | 9.68 | 9.82 |
| Chrysopidae | 10.69 | 13.78 | 16.40 | 7.67 | 16.25 | 15.95 | 18.48 | 12.27 | 7.67 | 14.60 | 6.22 | 11.02 | 12.09 | 6.05 | 5.57 |
| Coccinellidae | 22.74 | 26.57 | 29.03 | 36.08 | 53.95 | 22.75 | 21.17 | 22.09 | 28.02 | 28.47 | 23.27 | 28.15 | 20.75 | 25.74 | 34.72 |
| Geocoridae | 0.00 | 1.25 | 1.19 | 0.00 | 0.00 | 0.00 | 0.24 | 0.43 | 2.04 | 2.27 | 1.45 | 0.65 | 1.06 | 0.00 | 2.73 |
| Ichneumonidae | 2.45 | 2.38 | 1.79 | 2.41 | 2.35 | 4.65 | 1.43 | 1.66 | 3.58 | 1.46 | 2.22 | 3.24 | 2.18 | 1.87 | 1.82 |
| Pentatomidae | 0.72 | 1.13 | 1.26 | 2.56 | 2.43 | 2.29 | 2.30 | 5.70 | 3.58 | 2.19 | 4.52 | 1.39 | 5.05 | 7.81 | 6.38 |
| Syrphidae | 4.08 | 0.38 | 1.79 | 6.39 | 0.78 | 1.72 | 1.43 | 0.22 | 1.64 | 2.19 | 1.36 | 0.28 | 1.62 | 5.61 | 5.47 |
| Tachinidae | 24.91 | 14.54 | 17.39 | 12.78 | 7.04 | 25.68 | 27.99 | 14.22 | 18.30 | 16.46 | 29.33 | 20.83 | 24.36 | 18.70 | 15.89 |
| Vespidae | 0.91 | 2.38 | 0.73 | 1.70 | 0.52 | 0.64 | 2.06 | 1.37 | 1.84 | 2.11 | 1.53 | 1.39 | 2.24 | 5.83 | 1.82 |

# Figures

#
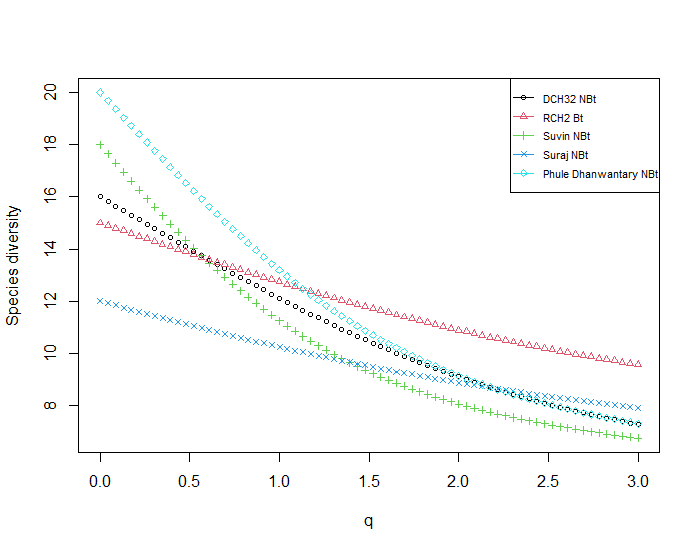


#
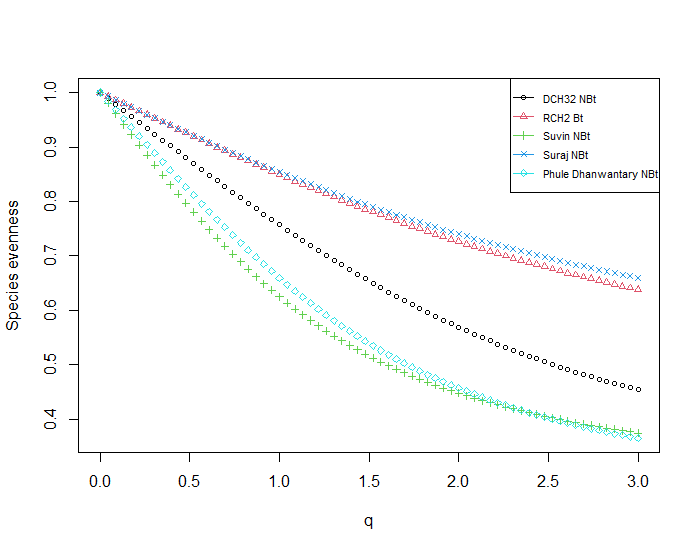


# Fig. S1. Species diversity and species evenness of natural enemies in the cotton test genotypes in 2016

#
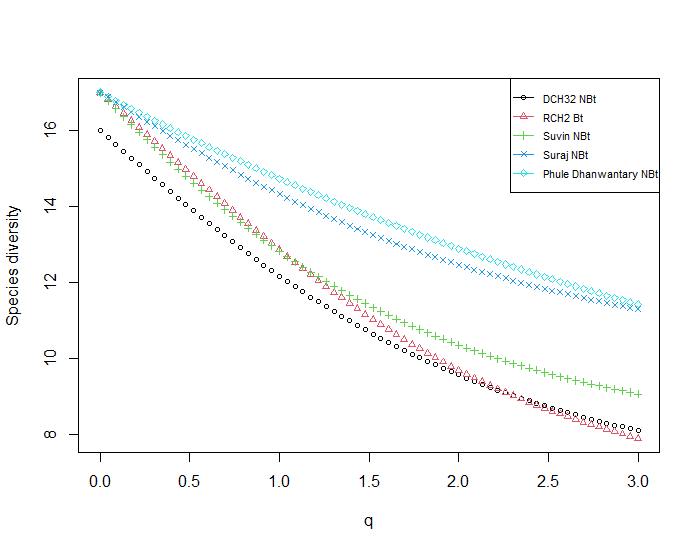


#
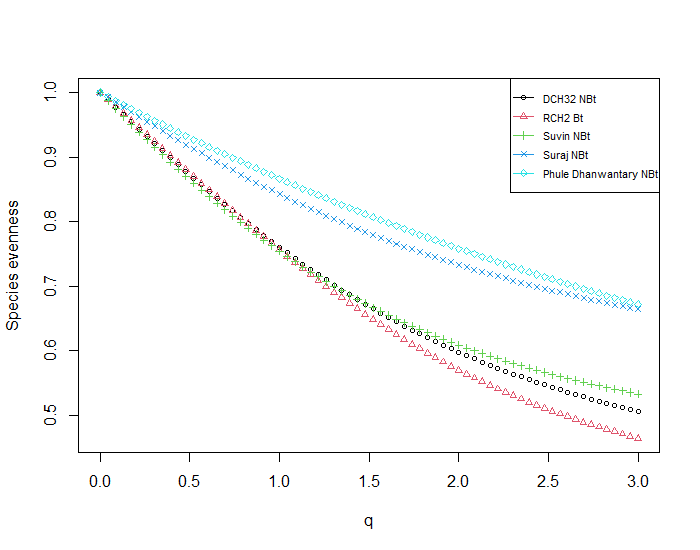


# Fig. S2. Species diversity and species evenness of natural enemies in the cotton test genotypes in 2017

#
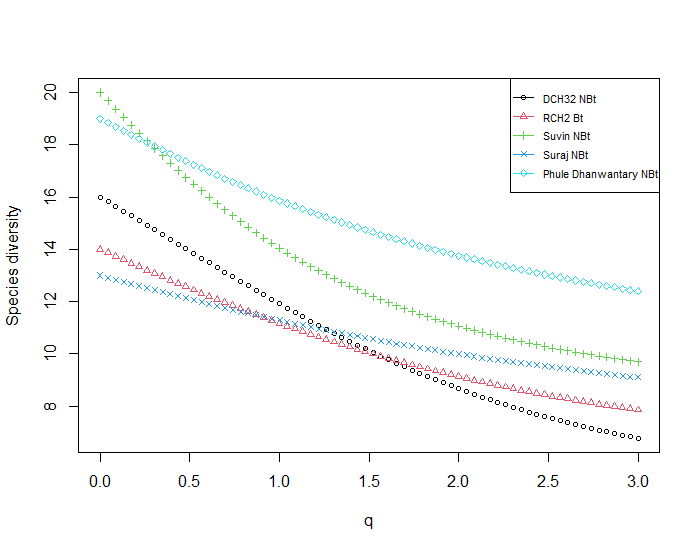


#
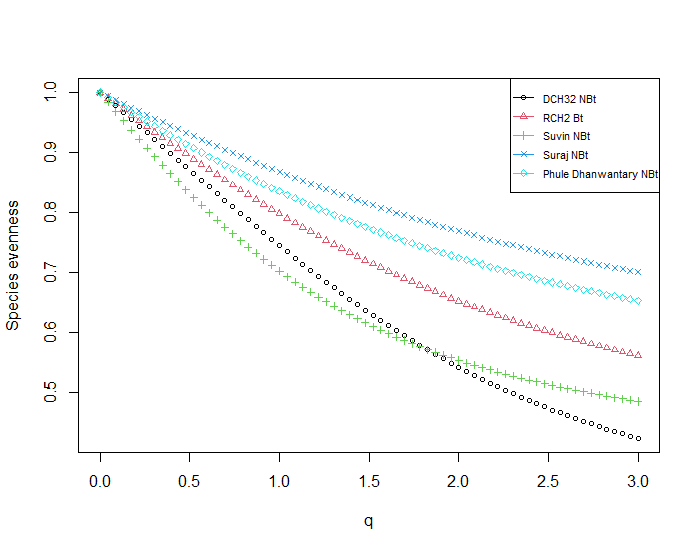


# Fig. S3. Species diversity and species evenness of natural enemies in the cotton test genotypes in 2018

#
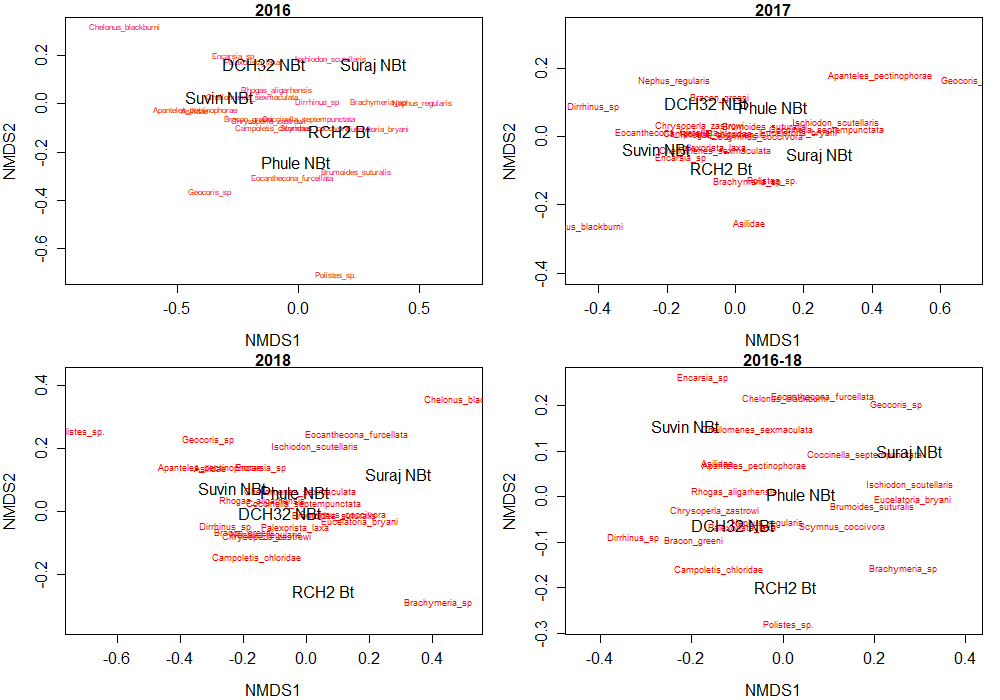


# Fig. S4. Nonmetric multidimensional scaling (NMDS) of the insect natural enemies of cotton test genotypes. Stress values for 2016, 2017, 2018 and 2016-18 are 0.071, 0.07, 0.077 and 0.071, respectively


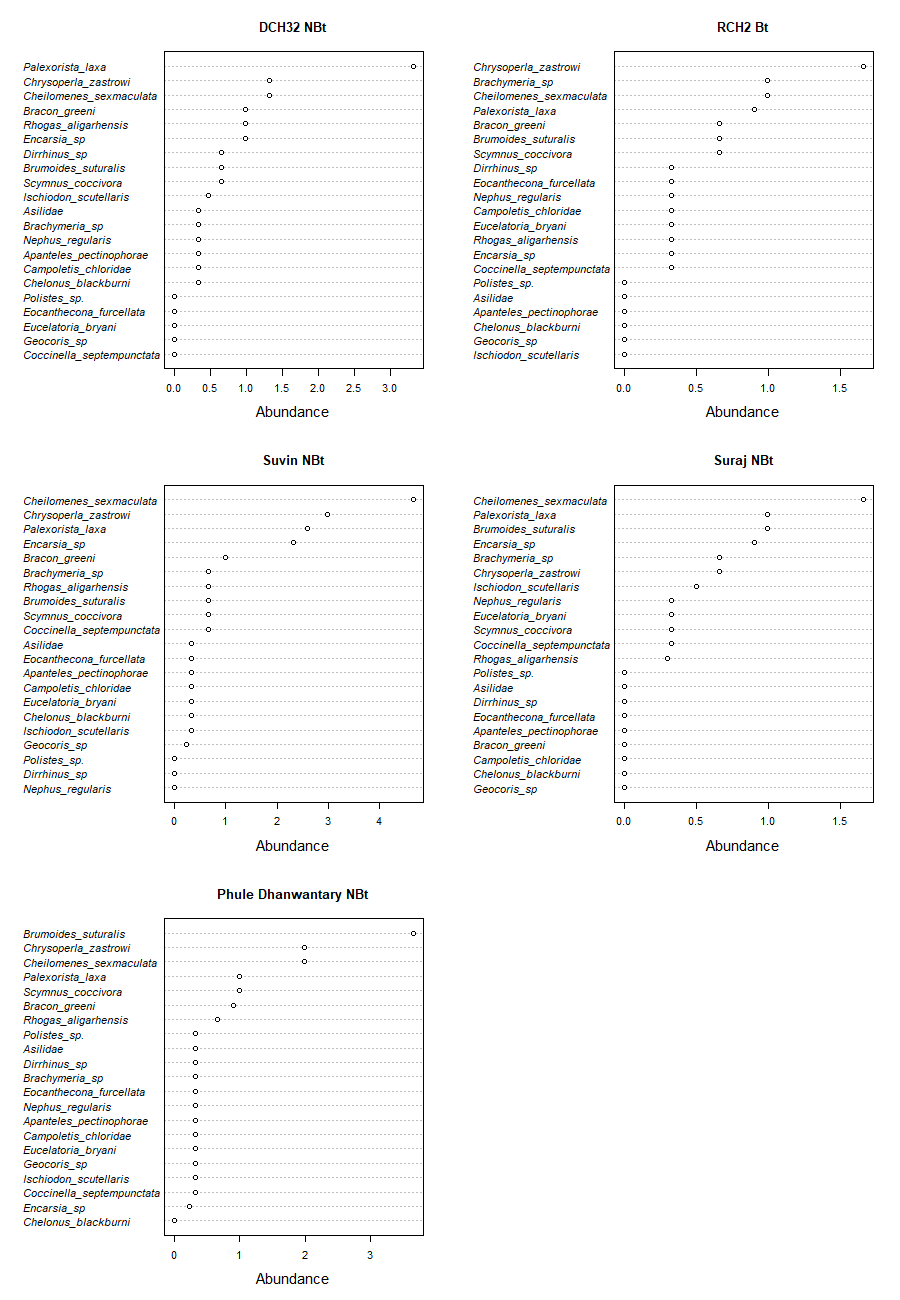


# Fig. S5. The seasonal mean of species abundance of natural enemies in the cotton test genotypes in 2016

#
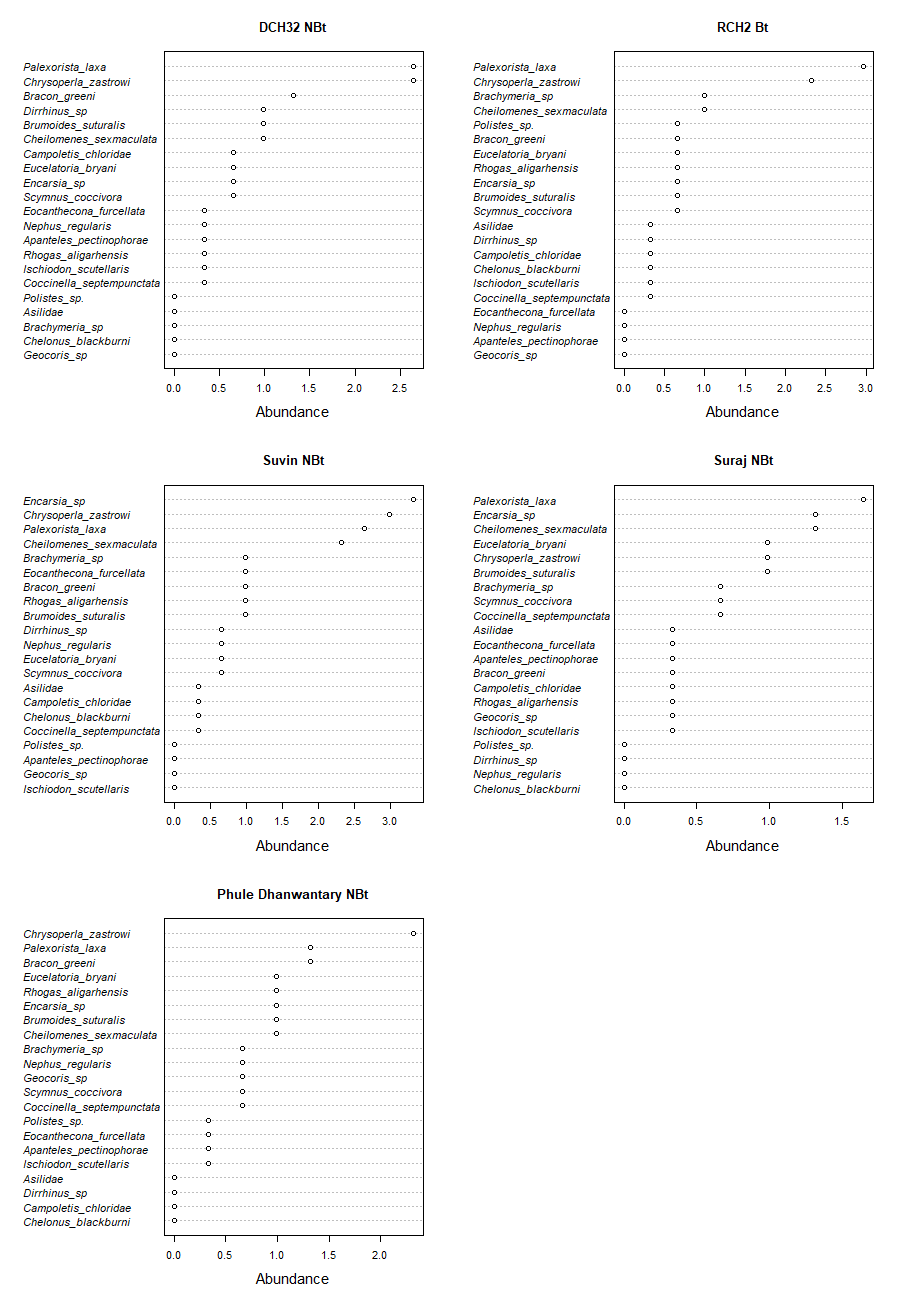


# Fig. S6. The seasonal mean of species abundance of natural enemies in the cotton test genotypes in 2017

#
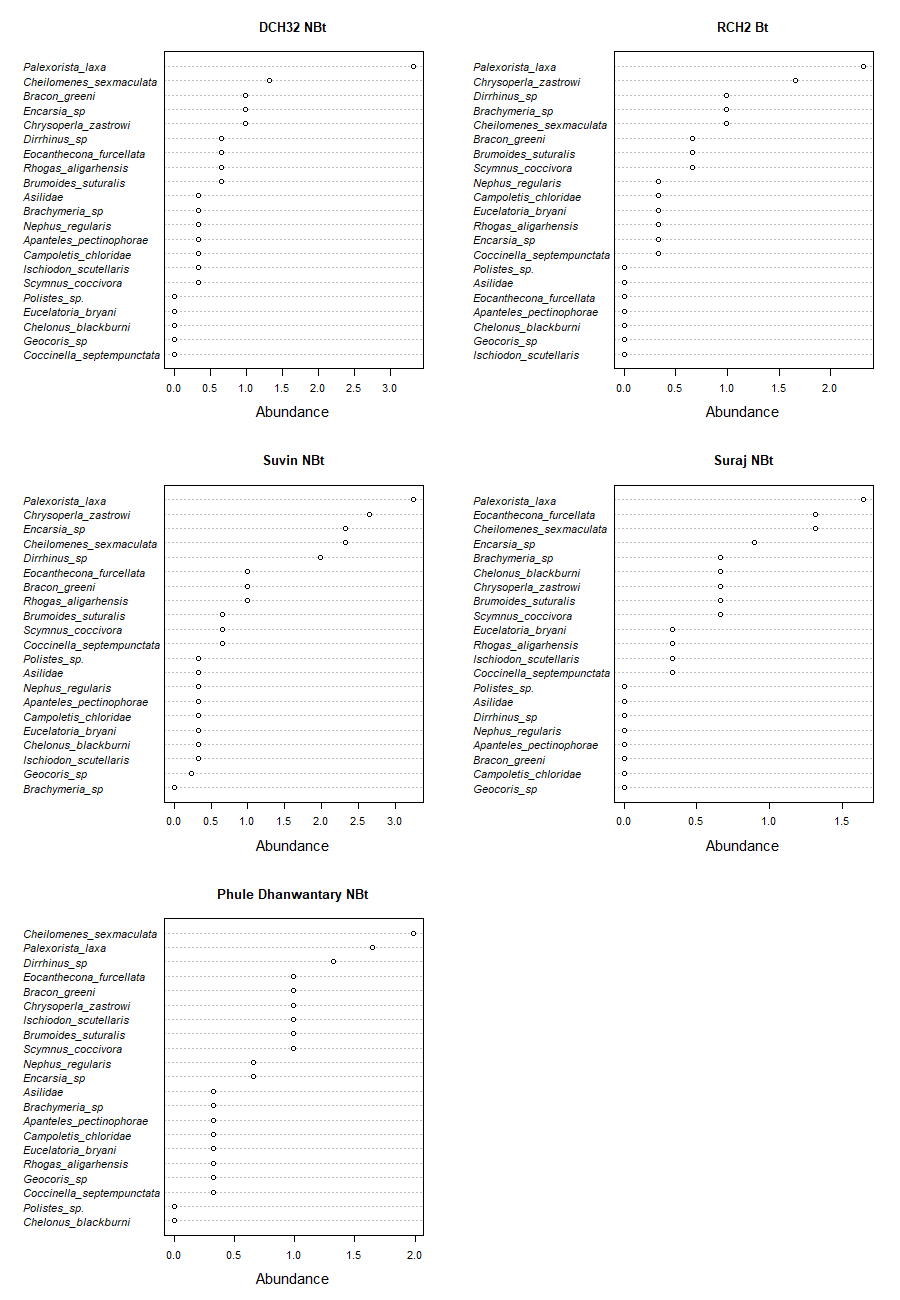


# Fig. S7. The seasonal mean of species abundance of natural enemies in the cotton test genotypes in 2018

#
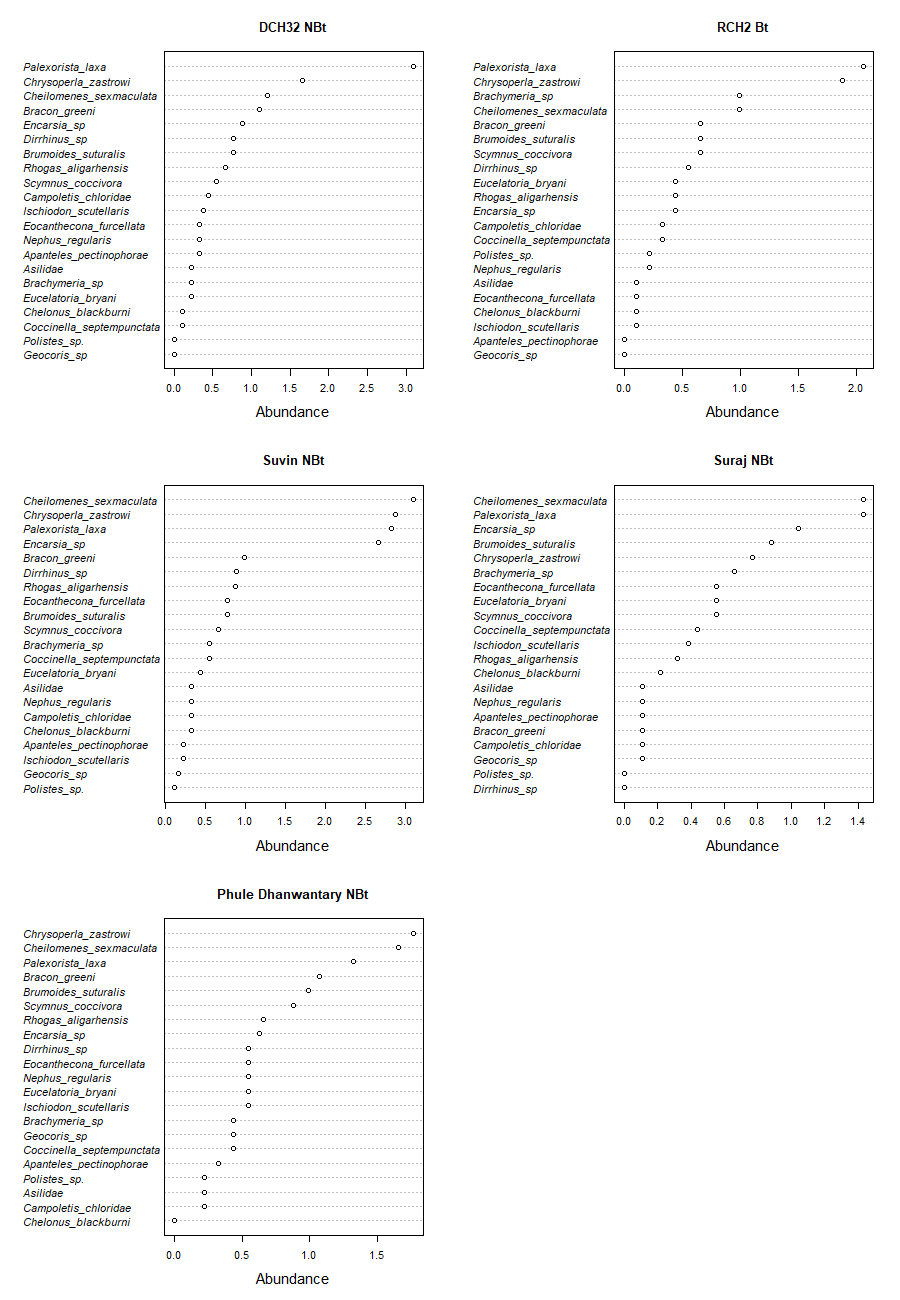


# Fig. 8 Species abundance of natural enemies in the cotton test genotypes for the pooled data (2016-2018)
